# N-acetylcysteine as add-on to antidepressant medication in therapy refractory major depressive disorder patients with increased inflammatory activity: study protocol of a double-blind randomized placebo-controlled trial

**DOI:** 10.1186/s12888-018-1845-1

**Published:** 2018-09-04

**Authors:** Chenghao Yang, Fokko J. Bosker, Jie Li, Robert A. Schoevers

**Affiliations:** 1 Tianjin Mental Health Institute, Tianjin Anding Hospital, No.13 Liulin Road, Hexi District, Tianjin, China; 2 University of Groningen, University Medical Center Groningen, Department of Psychiatry, Hanzeplein 1, 9700 RB Groningen, the Netherlands; 3 University of Groningen, Research School Behavioural and Cognitive Neurosciences (BCN), Hanzeplein 1, 9700 RB Groningen, the Netherlands

**Keywords:** N-acetylcysteine, Treatment resistant depression, Inflammatory activity, Biomarkers, Brain activity

## Abstract

**Background:**

A subgroup of depressed patients with increased inflammatory activity was shown to be more susceptible to develop Treatment Resistant Depression (TRD). Earlier studies with anti-inflammatory drugs have shown benefits in the treatment of major depressive disorder (MDD), but the effects are expected to be higher in patients with increased inflammatory activity. Supplementation of N-acetylcysteine (NAC) to ongoing antidepressant therapy may positively influence outcome of depression treatment in these patients. Therefore, this study aims to investigate the efficacy of NAC supplementation in patients with insufficient response to standard antidepressant treatment, and to explore potential roles of inflammation and oxidative stress involved in the alleged pathophysiological processes of TRD.

**Methods/design:**

A double-blind randomized placebo-controlled study comparing NAC versus placebo as add-on medication to antidepressant treatment with 12-week treatment and 8-week follow up in patients with TRD and increased inflammatory activity. Apart from clinical efficacy defined as the change in Hamilton Depression Rating Scale (HAMD)-17 score, secondary outcomes include changes in pathophysiological mechanisms related to depression as well as changes in local brain activity (functional Magnetic Resonance Imaging, fMRI) and white matter integrity (Diffusion Tensor Imaging, DTI). Importantly, sole patients with CRP levels with values between 0.85 and 10 mg/L will be included.

**Discussion:**

This is the first clinical trial taking both TRD and increased inflammatory activity as inclusion criteria. This study will provide reliable evidence for the efficacy of NAC in patients with TRD displaying increased inflammatory activity. And this study also will help explore further the roles of inflammation and oxidative stress involved in the alleged pathophysiological processes of TRD.

**Trial registration:**

The trial protocol has been registered on “ClinicalTrials.gov“with protocol ID “NAC-2015-TJAH” and ClinicalTrials.gov ID “NCT02972398”.

## Background

MDD is a highly recurrent, insidious and heterogeneous mental disorder. Worldwide the disease burden for both individuals and society is enormous [1], and although some progress has been made in the treatment of MDD, it has been estimated that more than one third of MDD patients do not respond satisfactorily to the initial and subsequent antidepressant treatments, including combinations of pharmacotherapy and psychotherapy [1]. Overall, 20–30% of patients with MDD have TRD, which accounts for a large proportion of the overall costs of MDD [2]. There is thus an urgent need to develop new and more effective therapeutic strategies.

Recent research indicates that both inflammatory processes and oxidative stress are part of the biological substrate of depression. It has also been suggested that neuro-inflammation plays a key role in TRD [3, 4]. Indeed, inflammation markers such as the acute phase protein CRP, pro-inflammatory cytokines, acute phase proteins and adhesion molecules are often increased in depressed patients. Notably, neuro-inflammation has been associated with structural/functional anomalies in the brain [5] but also with less propensity to respond to antidepressant treatment [6]. Furthermore, MDD patients with increased inflammation are more likely to have a chronic course despite antidepressant treatment [7]. This has led to the idea that additional anti-inflammatory treatment might improve treatment efficacy. A recent meta-analysis by Kohler et al. [8] indeed suggested that anti-inflammatory treatment, in particular celecoxib, has an effect on depressive symptoms thus advocating further studies in subgroups that could specifically benefit from such treatment. This is in line with an RCT using the TNFα inhibitor infliximab, which showed that in particular patients with increased inflammation had a significantly better response compared to placebo [9].

The glutathione precursor N-acetylcysteine (NAC) has been reported to positively interfere with several pathophysiological processes in MDD, including neuro-inflammation, glutamate neuronal activity, neurogenesis and apoptosis [10]. Preliminary evidence from a limited number of clinical studies indicates that NAC supplementation may also be beneficial in the treatment of other mental disorders such as schizophrenia, bipolar disorder and autism [11]. However, a recent randomized controlled trial in MDD could not demonstrate a statistically significant overall effect of NAC supplementation to regular antidepressant treatment at 12-week endpoint [10]. Yet, a secondary analysis suggested a positive effect of NAC in patients with more severe depression. In this study, inflammatory activity was not specifically assessed.

In summary, those studies described above on the relationship between anti-inflammatory agents and anti-depressive outcomes have specific strengths and weaknesses. Especially, no specific trial was conducted to study the efficacy of anti-inflammatory agents on the TRD patients with increasing inflammatory activity.

## Objectives

Our aim is to investigate antidepressant efficacy and safety of NAC in patients with TRD, displaying increased peripheral inflammatory activity and moderate to severe depression. We will also examine a range of biomarkers related to potentially important underlying biological mechanisms such as oxidative stress and inflammatory activity. Apart from studying the effects of NAC on depression severity, we will also study the effects on brain functioning (fMRI) and on white matter integrity (DTI).

## Methods

### Study design

This is a double-blind, randomized, placebo-controlled antidepressant augmentation trial. All participants are randomly divided into two groups treated orally with “antidepressant + NAC” (*N* = 100) or “antidepressant + placebo” (*N* = 100).

### Duration

Total study duration: 20 weeks (12-week treatment and 8-week follow up).

### Setting

Treatment will be given at the Tianjin Anding Hospital or at home. fMRI scanning is performed at the Imaging Center of the Tianjin Huanhu Hospital.

### Population (base)

The study population will consist of a sample of 200 patients with major depressive disorder recruited from the outpatient and inpatient departments of Tianjin Anding Hospital. The first 50 participants will be recruited for the MR scanning.

### Inclusion criteria

1. A current episode of MDD diagnosed according to Diagnostic and Statistical Manual of Mental Disorders, Fourth Edition (DSM-IV-TR) diagnosed with Structured Clinical Interview for DSM-IV (SCID)
2. Age between 18 and 65 years
3. A total score of 17 items Hamilton Depression Rating Scale (HAMD-17) ≥ 17
4. A CRP level between 0.85 and 10 mg/L
5. Insufficient response to one or more antidepressants given for at least 6 weeks and in an adequate dose during the current episode
6. Stable dose of the current antidepressant drug for at least 2 weeks prior to initiation of the study
7. Patients are compliant with treatment according to the judgement of the treating clinician.
8. Female subjects will be eligible to participate in the study if they are of non-childbearing potential or of child-bearing potential and willing to practice appropriate birth control methods during the study. Clinical patients always get a pregnancy test before start of treatment.
9. Participant or guardian has to sign informed consent. The patients’ guardians will sign the informed consent on behalf of the participants when the capacity of participants to consent is compromised.

### Exclusion criteria

1. A history of manic episode
2. Use of mood stabilizer
3. Use of antipsychotic medication with more than half of the maximum dosage suggested in the instruction
4. History of substance abuse or dependence
5. An allergic reaction to NAC or any component of the preparation
6. Severe somatic diseases that might interfere with regular antidepressant treatment including conditions such as kidney and liver failure, uncontrolled hypertension, cardiovascular, cerebrovascular and pulmonary disease, thyroid disease, diabetes, epilepsy and asthma.
7. Use of anti-inflammatory medication for longer than 7 days in the last 2 months preceding the trial
8. Use of immunosuppressive medication such as oral steroid hormones
9. History of chronic infection, such as tuberculosis, AIDS, hepatitis
10. CRP value > 10 mg/L
11. Women in pregnancy or lactation period

**Additional exclusion criteria** for patients participating in the MR scanning Participants have to fill out a detailed questionnaire covering safety aspects in relation to research in a 3 Tesla magnetic field and MRI environment. These criteria are:

1. MR incompatible implants in the body (such as cochlear implant, insulin pump, pace maker or other metal implants)
2. Any risk of having metal particles in the eye, due to manual work without proper eye protections
3. Tattoos containing red pigments
4. Claustrophobia
5. The refusal to be informed of structural brain abnormalities that could be detected during the experiment

### Sample size calculation

There are only few data available on effect size and standard deviations for NAC as an antidepressant, and power calculations therefore remain speculative.

One RCT gave 1000 mg NAC daily as add-on medication for TRD in a total of 75 included patients with bipolar disorder and found a statistically significant effect on the MADRS (depression severity) and Global Assessment of Functioning (GAF) outcomes [12]. A second RCT has treated 269 MDD patients with 2000 mg NAC augmentation daily for 12 weeks and found no statistically significant difference on the primary MADRS outcome in the whole group. However this study did show significant effects on the CGI and functional outcomes in patients with more severe depression who had an initial MADRS score > 24 (*p* < 0.03) [10]. In our study we also focus on patients with more severe depression.

For the power analysis we therefore assumed that in the placebo group 20% will have a positive outcome and 40% in the intervention group. Setting á at 0.05 and â at 0.80 in a two-tailed independent t-test indicated that a minimum of 91 patients per group is needed (a total of 182 patients with continuity correction). Considering 10% drop-out, we raised the inclusion to 100 per group, thus a total of 200 included patients.

### Strategy for participant enrolment

Firstly, a well-trained team is involved in the enrolment, including one senior psychiatrist, two research nurses, two master students working full time, and 20 psychiatrists motivated to refer patients for scientific research. Secondly, by giving a more elaborate explanation of the study goals, including the pros and cons, to both patients and their guardians they now better understand the background and possible relevance of the study. Still, financial support also assists to this work. Lastly, as Tianjin Anding hospital is the largest mental health facility in the north of China, with around 540,000 outpatients every year and 1300 beds, we are confident to finish the study on schedule using this adapted procedure.

### Study parameter/endpoint

#### Main study parameter/endpoint

HAMD-17: The primary outcome criterion is the change in HAMD-17 score between the pre-treatment and the first post-treatment measurement.

#### Secondary study parameter/endpoint

1. HAMD-17 at 8 weeks’ follow-up after discontinuation of NAC treatment
2. Beck Anxiety Inventory (BAI)
3. Inventory of Depressive Symptoms - Self-Rated (IDS-SR)
4. WHO Disability Assessment Schedule (WHODAS-II)
5. Montreal Cognitive Assessment (MoCA)
6. Neuroimaging: DTI and resting state BOLD in the anterior cingulate cortex (ACC) before and after treatment in order to determine the general effect on fiber integrity projected out from ACC and brain metabolism in ACC (for details see below).
7. Biomarkers representing different pathophysiological mechanisms associated with MDD are assessed in venous blood and morning urine. All will be correlated with the intervention category and with primary and secondary outcomes.

#### Side effects

Possible side effects will be tabulated based on participant reports throughout the trial using a Checklist of 52 Somatic Items (CSI) described by Berk et al. [12].

#### Additional study parameters

The influences of gender, age, medication, body weight, level of treatment resistance assessed by Dutch Method for staging Therapy-Resistant-Depression (DM-TRD) on the study outcome will be investigated.

#### Randomization, blinding and treatment allocation

Subjects will receive NAC or placebo treatments in a randomized controlled double-blind design. The investigators, clinicians, and statisticians are blind to treatment allocation until the data analysis is completed. This is accomplished as follows:

The placebo is matched to NAC in shape, smell and color and capsules are sealed in identical bottles.

The medication is labelled as trial medication and given a random trial number by the trial pharmacist from 1 to 200. The list with numbers is stored in a secure place and nobody apart from the trial pharmacist has access to it until the trial is finished.

1. A second person who is not involved in any aspect of the trial will perform the randomization procedure by generating a medication number based on a list with consecutive trial participants (also from 1 to 200). Randomization procedure: participants will be assigned to the experimental group or control group using a randomized procedure according to enrolled order. From a table of random numbers, the patient will obtain a number.
2. The nature and dose of the primary therapy is monitored.
3. Participants are interviewed separately and have no opportunity to share their experiences.

### Study procedures

#### Overview of procedure

**Fig. 1:**
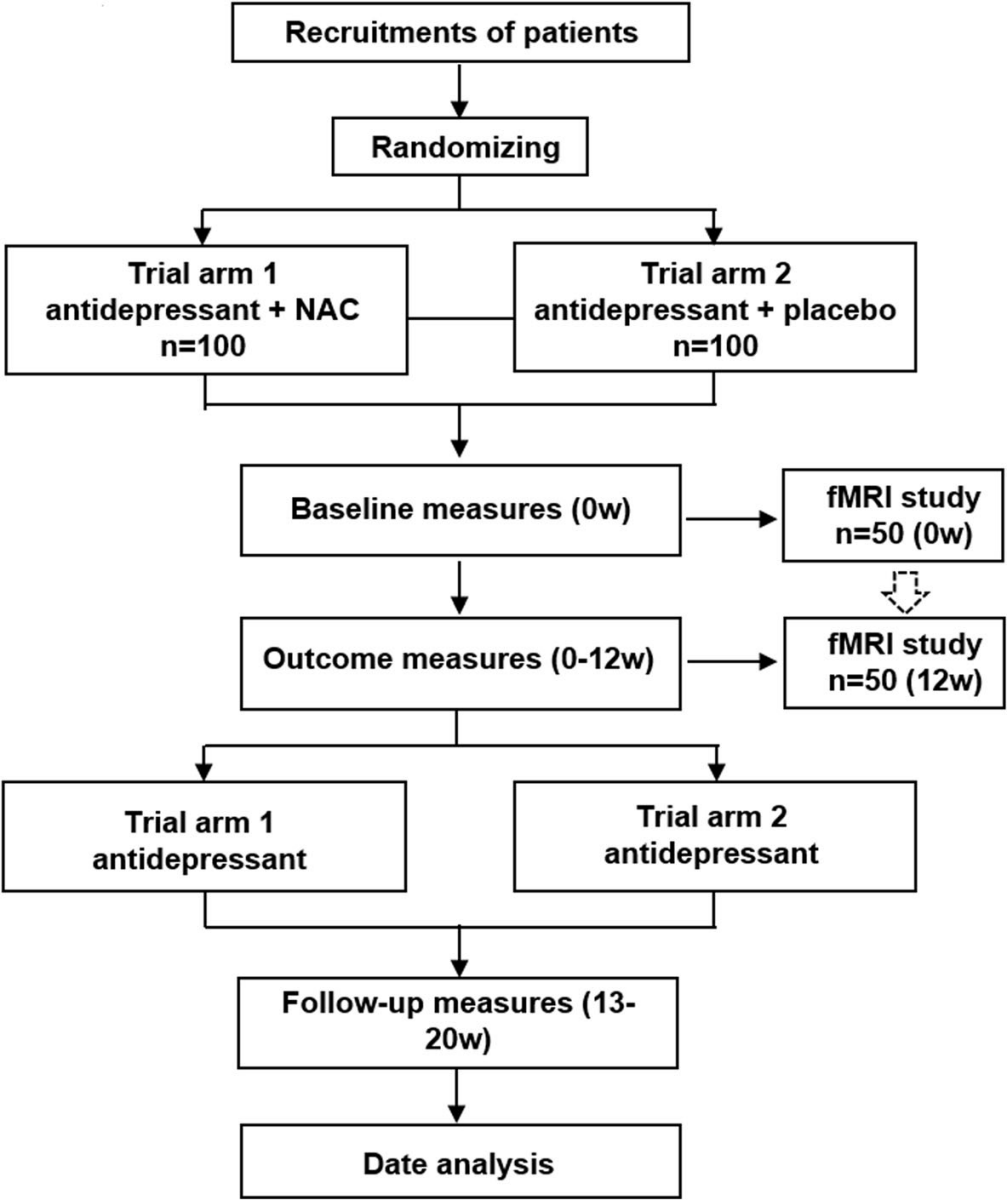
Trial flowchart. Eventually 200 patients will be required to finish the treatment study, being randomly divided into two groups including Trial arm 1 (antidepressant + NAC, *n* = 100) and Trial 2 (antidepressant + placebo, *n* = 100). For the fMRI scanning at baseline (week 0) and the 12th week, 50 patients will be included

Subjects receive care as usual while participating in this study, except that it is not allowed to change the antidepressant medication during the study. Antidepressants are restricted to selective serotonin reuptake inhibitors (SSRI) and serotonin and noradrenalin reuptake inhibitors (SNRI) and remain unchanged for at least 2 weeks before entering the trial (sertraline 50-200 mg/d, paroxetine 10-60 mg/d, citalopram 20-60 mg/d, venlafaxine 75-300 mg/d, duloxetine 30-90 mg/d). Benzodiazepines (BZD) are allowed to relieve anxiety in the first 2 weeks of antidepressant treatment and for sleeping problems during the trial if deemed necessary by the treating physician. BZD use will be recorded at all assessments during the trial and follow-up. It is not allowed to prescribe any antipsychotic medication with more than half of the maximum dosage suggested in instructions or as a mood stabilizer. The experimental NAC treatment is an addition to ongoing antidepressant treatment. During the study, it is not allowed to change medication (apart from BZD) or to commence ECT. (see Fig. 1).

#### Scale assessments

All scales mentioned above are used. See Table 1 for details.

**Table 1:** Scale assessments

Scale 1	HAMD-17	Self-rating scale for depressive symptoms
Scale 2	IDS-SR	Self-rated inventory for depressive symptoms
	BAI	Self-reported inventory for anxious symptoms
	CSI	A list for somatic items
	WHODAS-II	To assess functioning
	MoCA	To assess cognition
Scale 3	DM-TRD	To measure treatment resistance

#### Medication use

A dose finding study in six patients indicated that a NAC dose of 3000 mg daily caused intolerable stomach complaints. We therefore decided to use the same dose as in the Berk study [10]. Accordingly, all participants receive five NAC (200 mg) capsules twice daily (2000 mg daily) or matching placebo capsules in the 12-week treatment period, as add-on to the existing treatment for their major depressive episode. After the treatment period NAC/placebo is withdrawn, while during the 8-week follow-up regular treatment is kept the same.

#### Quality control

All selected scales are established, well validated and reliable. Two senior psychiatrists possessing ample experience with the SCID diagnostic scale will guarantee an accurate diagnosis and resolve any discrepancies during the scale rating. Moreover, all the professionals involved in scale rating have undergone a test on internal consistency with a kappa value of 0.81.

#### Monitoring adherence

Concentrations of NAC and its metabolites will only be measured in blood and urine at the different time points after the study being completed. It is therefore not possible to actively monitor adherence during the study in this way. However, pill counts for adherence are done by the pharmacy department, and an independent person confirms the capsule audit. Furthermore, we are confident that patients will adhere to the treatment with NAC through an intensive collaboration with the patients’ guardians as well as a financial encouragement for participants who complete the study.

### Routine recordings

#### Basic information

Name, gender, age, race.

#### Physical examination

Height, weight, waist circumference, waist-hip ratio, BMI, blood pressure, heart rate.

### Laboratory examination

#### Routine test

Blood and urine tests including measurements of hepatic and renal function, glucose, lipids and electrolytes.

#### Specific test

Blood (serum) and urine will be collected and stored after centrifugation at − 80°C. Samples will be used for a recently developed biomarker panel for depression supplemented with a number of inflammatory markers.

##### Specific test

Blood (serum) and urine will be collected and stored after centrifugation at − 80°C. Biomarkers in serum and urine will be measured using commercially obtained ELISAs displaying low cross reactivity. The serum and urine biomarkers are based on the monoamine, stress, inflammation and neuroplasticity/neurogenesis hypotheses of MDD supplemented with markers for metabolic syndrome, oxidative stress, endothelial dysfunction and magnesium deficiency. A combined biomarker panel has recently been developed and validated in collaboration with the University Medical Centre of Groningen (UMCG) in the Netherlands (UCP). Briefly, in a case control study a combined serum/urine biomarker panel for MDD with 80% sensitivity and 85% specificity was identified in the ROC analysis. A number of these biomarkers were replicated in a recent antidepressant augmentation study with the anti-oxidant curcumin [13]. The biomarkers in blood and urine are outlined below.

##### Blood

- Inflammatory: CRP; NGAL; IL6; TNF-α; TNF-α-R2; IFN-γ; S100a (p11); NF-ĸB; S100b
- Metabolic: insulin; leptin; vit D
- Growth factor: BDNF; VEGF; EGF^1^
- Stress: cortisol; arginine-vasopressin
- Neuromodulator/neuroplasticity: NPY, substance P
- Oxidative stress: c-GMP; isoprostane; SOD, CAT, glutathione, NO, MDA2
- Endothelial function: calprotectin; endothelin; zonulin
- Mineral homeostasis: aldosterone; thromboxane; cAMP

##### Urine

- Inflammatory: HVEM, LTB4
- Growth factor: EGF
- Stress: cortisol^1^
- Neuromodulator/neuroplasticity: substance P^1^, midkine
- Oxidative stress: c-GMP, isoprostane
- Endothelial function: calprotectin
- Mineral homeostasis: aldosterone^1^; thromboxane^1^
- Metabolic: leptin
- Miscellaneous: m-hydroxyphenylacetate, formate, alanine, creatinine, malonate, N-methylnicotinamide

(Note: ^1^ Those biomarkers were identified to change in a double blind augmentation study with curcumin by Lopresti, Maes et al. in Eur Neuropsychopharmacol 25(1): 38–50.)

### Lists of time points for examinations/assessments

#### 12-week treatment

Examinations and assessments during treatment include physical examination, scale assessments, medication use inventory, and laboratory examinations. All the time points are depicted in Table 2.

**Table 2:** clinical assessment time points

	Physical Examination	Scale Assessments	Medication use inventory	Laboratory examinations
Baseline	√	Scale 1, Scale 2, Scale 3	√	Routine test, Specific test
Week 2	√	Scale 1		
Week 4	√	Scale 1, Scale 2		
Week 6		Scale 1		
Week 8	√	Scale 1, Scale 2		
Week 10		Scale 1		
Week 12	√	Scale 1, Scale 2	√	Routine test, Specific test

Week 2 indicates that the time point is at the end of the second week after baseline, while other time points are synchronized in the same way

#### 8-week follow-up

Examinations and assessments in follow-up duration include physical examination, scale assessments, and laboratory examinations. All the time points are depicted in Table 3.

**Table 3:** Time points in 8- week follow up

	Physical Examination	Scale Assessments	Laboratory examinations
Week 14	√	Scale 1	
Week 16		Scale 1, Scale 2	
Week 18		Scale 1	
Week 20	√	Scale 1, Scale 2	Routine test, Specific test

Week 14 indicates that the time point is at the end of the 14th week after baseline, while other time points are synchronized in the same way

#### Neuroimaging

Patients undergo two MRI scan sessions. The first session takes place before the treatment starts while the second session takes place in week 12, before the study medication is terminated. During this procedure, participants will be exposed to a 3 Tesla magnetic field strength with switching gradient fields, radio waves and scanner noise.

An anatomical scan will be made to obtain the structural image necessary for the localization of functional activation. Patients will only be included in the study upon agreeing to be informed of any coincidental pathological findings. A statement to that effect must be signed before entrance into the study.

A functional MRI scan (either ASL or EPI) is recorded during the resting state, i.e. the subjects do not do any tasks during this scan. Breathing and heart rate are measured in order to remove variance that correlates with these rhythms.

A DTI scan is made with two fMRI tasks to assess connectivity changes due to the treatment. During the DTI scans, all using the EPI protocol, two fMRI tasks will be presented to the subjects.

1. Wall of faces [14]

A group of 32 emotional faces is presented at once to a subject. Subjects have to decide whether they perceived angrier or happy faces. As a baseline, subjects have to indicate if there are more males or females. The array of faces either contains an equal (ambiguous, 16:16) or unequal (unambiguous, 26:6) number of faces with one emotion/gender vs. the other. The duration of this task is 15 min.

2. Reward task [15]

We will use a monetary incentive delay task to investigate reward sensitivity in the two patient groups. We expect that both our groups will initially show aberrant activation patterns -as reward sensitivity is thought to be a state marker of anhedonia but that the intervention group will normalize during the course of the treatment. The task will be a modified version of the task described by Pizzagalli et al., [15] who found aberrant neural responses to both reward anticipation and experience in depression. Every trial consists of a cue indicating trial type (gain or neutral condition), target presentation and feedback. Subjects are told that on incentive trials they may gain money by pressing the button during target presentation. On neutral trials, subjects are not required to respond. The inter-stimulus-interval will vary to prevent expectancy effects. Cue presentation is known to elicit reward anticipation and feedback about gains elicits reward experience. Subjects will receive their obtained rewards at the end of the session. The order of feedback will be randomized and not associated with actual performance to ensure equal pay for all the subjects. The task will include 90 trials of 10 s each, so the total duration will be around 15 min.

#### Data management

All data will be entered online into OpenClinica (https://www.openclinica.com/). OpenClinica provides trace management of any adjustments in entered data, which warrants an effective data monitoring.

#### Auditing strategy

The sponsor will audit the proceeding of this study. Frequency and procedure of auditing comply with Good Clinical Practice (GCP) of China. This action is performed independently from the investigators.

### Safety reporting

#### Adverse events (AES)

Adverse events are defined as any undesirable experience occurring to a subject during the study, whether or not considered related to NAC. All adverse events reported spontaneously by the subject or observed by the investigator or his staff will be recorded.

#### Serious adverse events (SAEs)

A serious adverse event is any untoward medical occurrence or an effect that at any dose:

1. results in death
2. is life threatening (at the time of the event)
3. will require hospitalization or prolongation of existing inpatients’ hospitalization.
4. results in persistent or significant disability or incapacity

Any other important medical event that may not result in death, be life threatening, or require hospitalization, may be considered a serious adverse experience when, based upon appropriate medical judgment, the event may jeopardize the subject or may require an intervention to prevent one of the outcomes listed above.

#### Reporting of SAEs

The investigator shall report immediately the SAEs to the applicant and/or Clinical Research Organization (CRO, sponsor representative). The patient has to be unblinded urgently (two persons must be present) and receives emergency treatments. Within 24 h the investigator reports the SAEs to State Food and Drug Administration (SFDA) of China by faxing a SAEs form. These events also need to inform other concerned investigators.

#### Suspected unexpected serious adverse reactions (SUSARs)

Adverse reactions are all untoward and unintended responses to an investigational product related to any dose administered.

Unexpected adverse reactions are SUSARs if the following three conditions are met:

1. the event must be serious.
2. there must be a certain degree of probability that the event is a harmful and an undesirable reaction to the medicinal product under investigation, regardless of the administered dose.
3. the adverse reaction must be unexpected, that is to say, the nature and severity of the adverse reaction are not in agreement with the product information as recorded in: a) summary of Product Characteristics (SPC) for an authorized medicinal product; b) investigator’s Brochure for an unauthorized medicinal product.

#### Reporting of SUSARs

The procedure is same to the reporting of SAEs.

#### Unblinding procedure

Regular unblinding will take place after ending the treatment period by the research assistant. Emergency unblinding may only occur on an individual basis for safety reasons if the information can help the treatment of SAEs or SUSARs. For emergency unblinding, envelopes are available at the medication storage room at the in-patient ward, kept separately from the patches, for the 24 h unblinding service. In the case of a medical emergency situation as described below, the sealed envelope can be opened by anyone of the medical team and the treatment assignment of a participant will be unblinded. In case of an emergency unblinding, the reason and time of unblinding will be documented in the study files. The decision to unblind is at the discretion of the local investigator.

Emergency unblinding is indicated in the following situations only:

1. unblinding is necessary for the subjects’ emergency treatment at the local investigators discretion
2. unblinding is required by local laws or regulations (in case of SUSARs)

### Withdrawal

#### Withdrawal of individual subjects

Subjects can leave the study at any time for any reason if they wish to do so without any consequences. The investigator can decide to withdraw a subject from the study for urgent medical reasons. All withdrawals are considered as drop out and the reasons will be recorded.

#### Specific criteria for withdrawal

Withdrawal from the study will occur:

1. If participants cease taking their trial medication for seven consecutive days, stop effective contraception, or became pregnant.
2. If participants revoke their consent or develop serious adverse events associated with the study medication, which could occur either at the request of the patient or the discretion of the investigator.
3. If participants develop a manic episode.
4. If the antidepressant is modified during the trial – either by the treating physician or at the initiative of the patient.
5. If participants use non-steroid anti-inflammatory drugs (NSAIDs) during the trial for more than 7 days.
6. If participants use anti-oxidative substances in dosages above 200 μg of selenium/day or 500 IU of vitamin E/day.

#### Terminate participation

Reasons to terminate a patient’s participation include:

1. The patient withdraws her/his consent
2. Intolerance to the study drug: if a patient experiences unacceptable side effects, the patient may decide, or make a joint decision with the treating physician, to terminate study participation.

### Statistical analysis

#### Descriptive statistics

The data acquired from questionnaires and the other non-imaging data are summarised by the mean and standard deviation in case of normal distributions and by median and quartile in the case of non-normal distributions.

For the imaging data we will use Statistical Parametric Mapping 8 (SPM) (Wellcome Department of Cognitive Neurology London, UK; http://www.fil.ion.ucl.ac.uk). Pre-processing will be done on the data including realignment, co-registration, and spatial normalisation into a standard space and smoothing. Statistical analysis will be based on a voxel-by-voxel multiple regression analysis of the time-courses. First level analyses will be done using GLM. For the second level, random effects analysis will be conducted. Contrasts in a second level analysis will be made between the four study groups. Furthermore, correlations will be calculated between brain activation in a region of interest and questionnaire scores.

#### Analysis of treatment effect

The effect of treatment (placebo/NAC) is investigated on the HAMD-17 by comparing differences between both groups in change of severity scores between baseline and endpoint (12th). And we also compare differences between the baseline and other time points (2nd, 4th, 6th, 8th and 10th) and for 8 weeks follow-up (until week 20). Linear mixed models are used because these models have the advantage of using all available data. We will use models with treatment group, time since intervention (as a categorical variable with two levels), and the interaction between time and treatment group, with pre-treatment scores as covariates. In this model, a significant main effect for treatment group indicates an overall treatment effect over the entire study period. The interaction term is added to assess the effect at the different time points (post-treatment and follow up).

#### Moderation of treatment effect

To investigate the potential moderating effects of other factors (e.g. age, gender), two separate ANCOVAs will be used to investigate whether treatment differences at post-treatment and/or at follow up are moderated by gender, age, or treatment expectancy. To this end, the treatment variable (placebo/NAC), the potential moderator, and their interaction will be included in the model. Pre-treatment values are used as a covariate to correct for possible differences in baseline values. Each potential moderator will be tested in a separate model.

## Discussion

A recent clinical study has shown beneficial effects of anti-inflammatory co-medication with antidepressants in depressed patients with increased levels of peripheral inflammation [16]. This is in line with a study by Raison et al. [9] who performed a proof-of-concept study with a TNF antagonist in patients with TRD and showed improvement in patients with high baseline inflammatory biomarkers. These studies provided the rationale to design the current randomized controlled trial with the anti-inflammatory agent N-acetylcysteine in TRD patients with increased inflammatory activity. Although an earlier study by Berk et al. failed to demonstrate overall significant beneficial effects of NAC supplementation to regular antidepressant treatment at 12-week end point [10], a secondary analysis suggested a positive effect of NAC in patients with more severe depression. In contrast to the present study inflammatory activity was not specifically assessed in the study by Berk et al. [10] but work from our own group has shown that increased inflammatory activity is related to non-response in depressed patients treated with antidepressants [7]. By sharpening the inclusion criteria in the present study, we expect to reduce heterogeneity of the patient sample, diminish possible confounding effects by other medications and develop a tailored intervention for patients with mood disorder with inflammatory dysregulation who have do not respond to regular phases of antidepressant treatment. To this end, solely participants with relatively higher CRP levels with values between 0.85 and 10 mg/L will be included, representing around one third of all MDD patients who are treated in our hospital. In addition, patients are selected on basis of treatment resistance defined as an insufficient response to 1 or more antidepressants given for at least 6 weeks in an adequate dose during the current episode. Moreover, restrictions will be made with respect to additional medication use, such as benzodiazepines, anti-inflammatory agents and antidepressant medications with anti-inflammatory properties. With these measures we strive to select a more homogenous subgroup of MDD patients for whom NAC may indeed provide an effective adjuvant treatment strategy to reduce their depressive symptoms. MDD has been associated with functional brain abnormalities including aberrant brain activity and abnormal functional connectivity between brain areas [17, 18]. Yet, functional imaging and peripheral biomarker measurements in patients with TRD are relatively scarce. By introducing such measurements in the present study we hope to provide additional information regarding the pathophysiological processes involved in TRD including their relation with treatment response [19]. Conversely such information may be useful to verify or falsify our working hypothesis that inflammatory processes and oxidative stress play a pivotal role in this TRD sample.

There are some limitations of this design. Firstly, there is still lack of consensus on the definition of TRD. For instance, depression severity in patients who do not sufficiently respond to 1 antidepressant treatment and patients not sufficiently responding to more than 1 antidepressant treatment may be significantly different, potentially causing a dysbalance between these groups. Similarly, our protocol does not take duration of the current episode into account, which may also be an aspect of treatment resistance [20, 21]. Secondly, putative confounding factors such as frequency of episodes, family history, and personality traits are not represented in the inclusion criteria. Thirdly, we choose a dose of 2000 mg NAC based on the results of earlier studies [10, 11], but it may be that a higher dosage is more effective. One study in ADHD showed beneficial effects with a dosage of 4800 mg [22]. However, decreased tolerability of this higher dose was clearly shown in our pilot study using 3000 mg. It should be noted that earlier studies with NAC in psychiatry were performed in Australian patient populations. Arguably Chinese people may be more sensitive to NAC administration as might be concluded by the beneficial effects of relatively lower dosages in chronic obstructive pulmonary disease (COPD) compared with Caucasians [23–25]. In addition, we now focus specifically on a TRD patient group with increased inflammatory activity which potentially may show a better response to treatment with an anti-inflammatory agent. Finally, the treatment duration we chose in our study is similar to that of previous studies [10] in which effects were found in the follow-up period but not at the end of treatment. For this reason, we prolonged the follow-up duration to 20 weeks. It is, however, conceivable that treatment for a longer period of time would yield better results.

In conclusion, despite these limitations, this study is expected to provide reliable evidence for the efficacy of NAC in patients with TRD displaying increased inflammatory activity.

## Abbreviations

ACC: anterior cingulate cortex
AES: adverse events
BAI: Beck Anxiety Inventory
BZD: benzodiazepines
CRO: Clinical Research Organization
CRP: C-reactive protein
CSI: Checklist of 52 Somatic Items
DM-TRD: Dutch Method for staging Therapy-Resistant-Depression
DSM-IV-TR: Diagnostic and Statistical Manual of Mental Disorders, Fourth Edition
DTI: diffusion tensor imaging
ECT: electroconvulsive treatment
fMRI: functional Magnetic Resonance Imaging
GAF: Global Assessment of Functioning
HAMD-17: Hamilton Depression Rating Scale-17
IDS-SR: Inventory of Depressive Symptoms - Self-Rated
MDD: major depressive disorder
MoCA: Montreal Cognitive Assessment
NAC: N-acetylcysteine
SAEs: serious adverse events
SCID: Structured Clinical Interview for DSM-IV
SFDA: State Food and Drug Administration
SNRI: serotonin and noradrenalin reuptake inhibitors
SPM: Statistical Parametric Mapping
SSRI: selective serotonin reuptake inhibitors
SUSARs: Suspected Unexpected Serious Adverse Reactions
TRD: treatment resistant depression
WHODAS-II: WHO Disability Assessment Schedule
